# Ziemssen’s Cyclopædia, Vol. VIII

**Published:** 1877-10

**Authors:** 


### Books Reviewed.

*Cyclopædia of the Practice of Medicine.* Edited by DR. H. VON ZIEMSSEN, Professor of Clinical Medicine in Munich, Bavaria. Vol. VIII. Diseases of the Chylopœtic System. New York: Wm. Wood & Co., 1876.

Diseases of the Naso-Pharyngeal cavity and pharynx are treated of by Prof. Hermann Wendt, of Leipsic, translated by J. Solis Cohn, M. D., of Philadelphia, in that exhaustive and philosophical manner so peculiar to the German intellect. The remarks upon the general anatomy of the regions is short but condensed and sufficient for a clear understanding of their relations and natural appearance, while the remainder of the article is full of interest.

Prof. W. Leube, of Jena, presents the diseases of the stomach and intestines, translated by Arthur Van Harlingen, M. D., and A. B. Ball, M. D. Dr. O. Leichtenstern, of Tubingen, writes up constrictions, occlusions and displacements of the intestines, which is translated by Lewis A. Stimson, M. D. Prof. Arnold Heller, of Kiel, treats of intestinal parasites; Dr. H. von Ziemssen, diseases of the larynx; and Dr. A. Steffen, of Stettin, spasm of the Glottis, which were translated by A. V. Macan, M. D., and E. W. Schauffler.

The subject of occlusion of the intestines, being one of interest at the present moment, on account of a recent case to which the attention of the profession has been called, see Art. II, we read with care the article by Dr. Lichtenstern, but were unable to find an account of any similar case. We were struck, however, with the lucid and thoroughly scientific manner in which the pathology and diagnosis of the various lesions of the intestines have been presented. It is a masterly essay on a subject of much interest to the profession, and although the treatment is so often unsatisfactory, even when diagnosis is sure, yet we must ever welcome anything which may be called an addition to our knowledge of the obscure.

Prof. Heller's article on intestinal parasites is readable and of considerable importance. The natural history of the various parasites are givon, together with the history of their discovery. The ovum and complete animal is truthfully shown by many illustrations. Dr. von Ziemssen is to be congratulated upon the manner in which he handles diseases of the larynx. This interesting subject is most exhaustively treated by the learned doctor, while the illustrations are such that salient points are easily grasped, especially in his description of the neuroses of the larynx. In regard to treatment of neuroses he very highly recommends electricity. He says: "The action of localized electricity is sometimes most brilliant, especially in hysterial paresis of tension. The aphonia, which may, perhaps, have lasted for months, not unfrequently disappears during the first sessions, and the normal voice takes its place. The spectacle, which strikes the layman as almost miraculous, of a hysterical patient, who for a long time has been quite voiceless, speaking only in a whisper, leaving the physician's consulting-room or electricity-room with a loud, ringing voice, is neither rare or difficult to understand." He, however, goes on to say that unfortunately the first result is not lasting, and frequent relapses often causes the treatment to drag along for months. Yet he seems to think it will succeed if anything is of use, always, unless the nerve lesions are intense. Perhaps we are skeptical, still, we cannot so entirely rely upon electricity as many others, and only hope for cure in a part, not in every case. The translator's note upon the use of the electric current in these cases, is of much practical importance if one would wish to follow Ziemssen.

On the whole, this volume is one that will increase the value of the series, and will be read with much interest. C.

